# Barriers to Mental Health Care Transition for Youth and Young Adults with Intellectual and Developmental Disabilities and Co-occurring Mental Health Conditions: Stakeholders’ Perspectives

**DOI:** 10.1007/s10597-024-01262-x

**Published:** 2024-04-15

**Authors:** Christine B. Mirzaian, Alexis Deavenport-Saman, Sharon M. Hudson, Cecily L. Betz

**Affiliations:** 1 Division of General Pediatrics, Children’s Hospital Los Angeles, 4650 Sunset Blvd. MS #76, Los Angeles, CA 90027 USA; 2 Keck School of Medicine of the University of Southern California, Los Angeles, CA USA; 3 AltaMed Institute for Health Equity, AltaMed Health Services, Los Angeles, CA USA

**Keywords:** Intellectual and developmental disabilities, Transition, Mental health care, Barriers to care

## Abstract

Youth and young adults (YYA) with intellectual and developmental disabilities (IDD) have high rates of co-occurring mental health (MH) conditions. The time during transition from pediatric to adult health and mental health care can be a very challenging, with risk of loss of services leading to poor outcomes. This study aimed to explore barriers to transition from pediatric to adult health and mental health care and services for individuals with IDD and co-occurring MH conditions, by eliciting the view of stakeholders, including disability advocates. Qualitative analysis was conducted using grounded theory, and themes were coded based upon the social-ecological model (SEM). We generated themes into multiple levels: the individual level, the family level, the provider level, the systems of care level, and the societal level. Stakeholders expressed a critical need to improve coordination between systems, and to increase provider availability to care for YYA with IDD and co-occurring MH conditions.

## Introduction

Children and adolescents with intellectual and developmental disabilities (IDD) experience high rates of co-occurring mental health (MH) conditions (Einfeld et al., 2006; Emerson & Hatton, 2007). Pooled prevalence of psychiatric conditions of individuals with intellectual disabilities (ID) are as high as 32% (Mazza et al., 2020), and similarly, prevalence of co-occurring MH conditions in individuals with autism are greater than in the general population (Lai et al., 2019). In addition, individuals with IDD are at increased risk of MH disorders during the period of transition to adulthood (Young-Southward et al., 2017a, 2017b, 2017c).

Transition of mental health (MH) care from pediatric to adult services can lead to discontinuity of care and leave youth vulnerable to adverse mental health outcomes (Cleverley et al., 2020). Transition of MH care appears to be equally, if not more problematic, than in physical care settings (Pinals et al., 2022; Singh & Tuomainen, 2015; Young-Southward et al., 2017a, 2017b, 2017c). Qualitative studies on transition in individuals with IDD have noted it can be “objectively terrifying” to youth and worrisome for their families (Cleverley et al., 2020; Young-Southward et al., 2017a, 2017b, 2017c). However, there is limited information about what makes this particular transition such a difficult process. Recent systematic reviews on transition in individuals with IDD indicated a gap in the literature in this area and a need for further studies (Brown et al., 2019; Reale & Bonati, 2015; Young-Southward et al., 2017a, 2017b, 2017c). Furthermore, the need for adequate health care transition support (HCT), especially for youth and young adults (YYA) with long-term conditions, has become widely acknowledged. The need for this service model was first introduced nearly 40 years ago by national leaders in pediatric, adolescent, and public health care (Blum et al., 1993; McGrab & Millar, 1989). At the time, the emphasis was focused on YYA with chronic medical illnesses. Over time, governmental system of care oversight increased, and the term YYA with special health care needs (SCHN) emerged (McGrab & Millar, 1989). Given this new call to action to healthcare leaders, educators, and researchers, the focus shifted towards service systems that were closely aligned with the pediatric system of care, wherein children and adolescents with SCHN received services from major pediatric medical centers, and pediatric practice networks. In response, pediatric major medical centers and practice networks began to develop and implement new HCT care models for YYA with specialized and complex medical needs. However, YYA, including many with IDD, access their care in community-based settings that do not offer emerging evidence-based HCT care, and therefore many of their health care needs may be overlooked.

As the pediatric service system was ramping up to develop and implement new HCT models of care, the focus was on the population of YYA with specialized and complex medical needs. This programmatic system of care emphasis created untoward consequences for YYA who were not directly involved with this service system. YYA with IDD, and those with MH conditions were often served in community-based settings that were not closely aligned with pediatric spheres of influence wherein HCT models of care were being developed and implemented. As a result, the practice and research in the field of HCT was not focused on populations of YYA with IDD, especially for those with co-occurring MH conditions (Culnane et al., 2021; Cvejic et al., 2018).

Unfortunately, this lack of inclusion in early HCT efforts is consistent with a greater health care disparity that exists among individuals with disabilities, which can be conceptualized as an unrecognized health care disparity population (Krahn et al., 2015), in part due to historical discrimination against those with disabilities as well as exclusion from society (Krahn et al., 2015). Despite major legislative efforts including the Americans with Disabilities Act (ADA) in 1990 and ADA Amendments Act of 2008, mandating equal access to health care for individuals with disabilities, negative, discriminatory, attitudes toward people with disabilities by health care providers can cause important barriers to health care (Lagu et al., 2022), making HCT an even greater challenge.

As the HCT field matures, attention is being directed not only to the development and implementation of evidence-based practice HCT models, but other populations of YYA whose need for services have been overlooked. Attention and efforts are now being directed to addressing the HCT needs of youth and young adults with IDD and those with co-occurring MH conditions (Brown et al., 2019; Culnane et al., 2021; Pinals et al., 2022). This study aimed to elicit existing barriers during transition for individuals with IDD with and without co-occurring MH conditions from the perspective of stakeholders, including interdisciplinary service providers, community-based stakeholders, parents and self-advocates.

## Methods

Participants were stakeholders associated with our University Center for Excellence in Developmental Disabilities (UCEDD) mailing list, which included internal UCEDD faculty and staff, as well as external collaborators who had chosen to subscribe to our mailing list. The mailing list contains representatives from our center’s UCEDD, other UCEDDs, other academic centers, as well as advocacy, and/or community-based organizations, consumers, and family members.

Data were collected using an anonymous and HIPPA compliant REDCap (Harris et al., 2009, 2019) electronic survey and database, between February and March of 2022. Participation was voluntary with no monetary compensation offered. The study was granted exempt status by our IRB.

Both quantitative and qualitative data were collected based on responses to close-ended and open-ended questions respectively. The *Survey on Transition Needs of Youth and Young Adults with Intellectual and Developmental Disabilities* used for this study was designed to elicit responses about the experience of individuals transitioning from child to adult health and mental health care for individuals with IDD. The items included in this survey were based upon the clinical expertise and experience of the research team and the existing body of literature (Betz & Coyne, 2020; Betz et al., 2021; Cheak-Zamora et al., 2022; Fair et al., 2016; Singh & Tuomainen, 2015; White et al., 2018). A brief version of the survey was piloted to evaluate understandability of the items with a sample size of 56 participants for an internal needs assessment. Open ended questions were then added to our final survey for this study.

All members of our UCEDD email list were sent an email invitation to participate in the survey. The survey was sent to approximately 3000 email addresses (the exact number was unable to be ascertained due to some email addresses being incorrect or no longer active). Minors (individuals under 18) were not allowed to participate. There were 283 initial respondents, however, 6 were not eligible due to being less than 18 years of age. In order to identify respondent type, our survey provided 3 choices to the prompt “Please chose the category that best describes you” 1. Provider (psychologist, psychiatrist, DBP, general pediatrician, nurse, social worker, etc.), 2. Stakeholder in community-based organization/resource (Regional Center [IDD service center], education, vocational rehab, etc.), or 3. Disabilities advocate (self-advocate, family advocate, professional advocate, etc.). Participants could each only choose one category for the respondent type that they most closely identified with. The total final sample size was 277. Quantitative results of this study will be published separately. The number who responded to the open-ended question “What other barriers exist for young people with IDD and mental health conditions in transitioning to adult mental health care?” was 105.

For qualitative analysis, responses to the open-ended question were entered into NVivo (Version 20) software (NVivo, 2020) and analyzed by three investigators using grounded theory approach (Corbin & Strauss, 2008) to analyze emerging themes. Grounded theory is a specific methodology developed for the purpose of building theory from data (Corbin & Strauss, 2008). The investigators chose this method as we did not wish to enter the analysis with a pre-determined framework, but rather develop one based on the data obtained. Investigators used an iterative process wherein participants’ responses were initially coded using an open coding approach, in which the data was broken apart and delineated into concepts, or codes to stand for raw blocks of data (Corbin & Strauss, 2008). We used axial coding and then grouped these concepts with similar themes (Corbin & Strauss, 2008; Gale et al., 2013; Strauss & Corbin, 1998). As demonstrated in Table 1, the three investigators each generated an initial code book, then discussed together to generate a code book based on consensus. The data was then re-coded with the agreed upon code book. The three reviewers then discussed the data after it was re-coded with the consensus code book and generated the resulting salient themes and began to see patterns that fit into the constructs of the social-ecological model (SEM) (Bronfenbrenner, 1977; McLeroy et al., 1988). The SEM had not been a model the authors had initially intended to use (as we did not enter the analysis with a pre-determined framework), but instead found that the themes naturally fit into this model. The constructs of the SEM were used to further organize the themes using selective coding (Corbin & Strauss, 2008), in which all codes were then placed into the most appropriate level of the SEM based on group consensus.

**Table 1:** Initial Codes and Consensus Codes

Investigator 1 Codes	Investigator 2 Codes	Investigator 3 codes
Access	Lack of access to care	Communication
Advocacy	Lack of information	Discrimination
Care coordination	Lack of knowledge and expertise	Foster Care
Culture	Lack of resources	Guardian
Discrimination	Language	Lack of MH providers or staff
Family Support	Mental health	Lack of patient or percent-centered philosophy
Family Training	Mental illness	Lack of resources
Foster care youth	Needs	Lack of transition planning
Housing	Parent	Lack of understanding or knowledge of difference between IDD and MH
Information overload	Providers	Language and culture barriers
Knowledge	Resources	Legal
Language	Services	Need for navigation, linkage and access support
Legal		Need for parent, consumer training, better information
Medication		Problems with mental health treatment
Mental health needs		Problems with self-advocacy
Professional training		
Resources		
Respect		
Self-Advocacy		
Sensitivity		
Time		
Timely Planning		
Transportation		
Wellness		
**Consensus Codes**		
Problems with access to care		
Self-advocacy		
Need for care coordination/ navigation		
Discrimination		
Need for family support/ training		
Legal issues		
Guardianship		
– Specific issues with foster care youth		
Need for resources		
– Housing		
– Transportation		
– Wellness		
Language and cultural barriers		
Problems with MH treatment		
– Medication over therapy		
Problems with transition planning		
– Timeliness		
Lack of MH providers/staff		
Lack of person-centered philosophy		
Lack of knowledge		
– Specifically, difference between MH and IDD		
Need for professional training		

## Results

As presented in Table 2, a total of 105 respondents completed the *Survey on Transition Needs of Youth and Young Adults with Intellectual and Developmental Disabilities* and responded to the open-ended question that was analyzed for this study. Disability advocates represented the highest number of respondents (n = 52; 49.5%), followed by community-based organization (CBO) representatives (n = 34; 32.4%), and providers (mental and physical health) (n = 19; 18.1%).). Each respondent chose only one category that they most closely identified with (total by type of respondent added up to n = 105) and had to chose one category in order to participate. Many respondents reported working with individuals with IDD across the life span, with the majority serving the age range of 19 to 21 years (n = 80; 76.2%). Racial/ethnic distribution of people with IDD served by rank order were Hispanic/Latinx (n = 84; 80%), White (n = 77; 73.3%), Black/African American (n = 77; 73.3%), Asian (68; 64.8%), Native Hawaiian/Pacific Islander (n = 83; 31%), American Indian/Alaska Native (n = 77; 28%) and Other (n = 27; 25.7%). Over 60% of respondents identified serving each of the diagnostic IDD groups listed with people with ASD (n = 96; 91.4%) and ID (n = 93; 88.6%) being most frequently identified. Of those who answered the open-ended question, 95.2% indicated that they served individuals with IDD and co-occurring MH conditions.

**Table 2:** Demographics and Characteristics of Populations Served

Characteristic	N = 105
	n (%)
Stakeholder type	
Provider	19 (18.1)
Community-based organization/resource	34 (32.4)
Disabilities advocate	52 (49.5)
Serve populations in California	
No	4 (3.8)
Yes	100 (96.2)
Activities carried out in stakeholder role	
Diagnostic testing	8 (7.6)
Direct health care (excluding mental health)	17 (16.2)
Direct mental health care	12 (11.4)
Psychosocial support	28 (26.7)
Empowerment and advocacy	79 (75.2)
Case management	48 (45.7)
Service referrals	59 (56.2)
Patient education	50 (47.6)
Age groups of people with IDD served	
5 years or less	69 (56.2)
6–10 years	61 (58.1)
11–15 years	68 (64.8)
16–18 years	71 (67.6)
19–21 years	80 (76.2)
22–26 years	71 (67.6)
27 years or older	60 (57.1)
Race/ethnicity of people with IDD^a^ served	
American Indian or Alaska Native	32 (30.5)
Asian	68 (64.8)
Black or African American	77 (73.3)
Hispanic or Latinx	84 (80)
Native Hawaiian or other Pacific Islander	40 (38.1)
White	77.(73.3)
Other	27 (25.7)
IDD or diagnoses of the populations served	
Autism	96 (91.4)
Cerebral palsy	76 (72.4)
Down syndrome	70 (66.7)
Epilepsy	71 (67.6)
Intellectual disability	93 (88.6)
Other	49 (46.7)
^b^IDD + MH	
No	5 (4.8)
Yes	100 (95.2)

^a^IDD = Individuals with developmental disabilities

^b^IDD + MH = IDD with co-occurring mental health conditions

Note: Some percentages add up to more than 100, as participants were able to select more than one option for some items. Participants could choose whether respond to questions, so data may be missing for some items

**Fig. 1:**
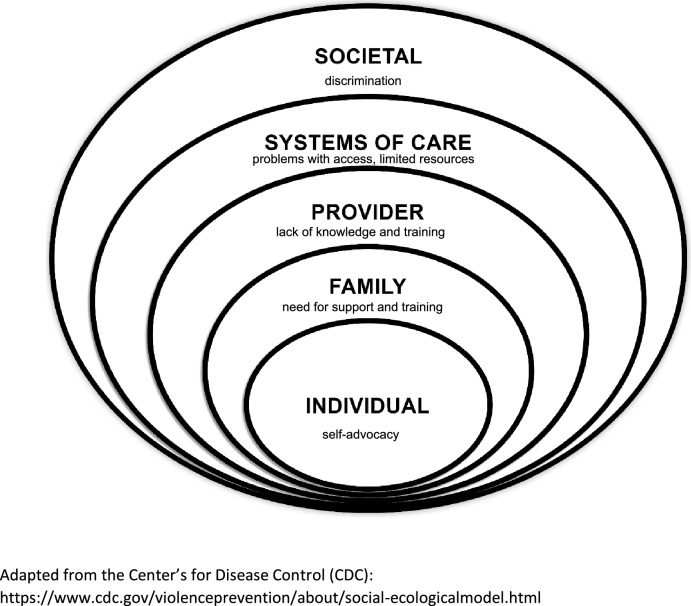
Barriers to transition of individuals with IDD and co-occurring MH conditions in the social ecological model

Barriers to adult care transition for YYA with IDD and co-occurring MH conditions were classified into 5 SEM levels: individual, family, provider, systems of care, and society, which are further explored below (Fig. 1):

## Individual-Level Barriers

Respondents reported barriers associated with the transition process to adult health and MH services that were associated with the knowledge and skills needs of young people with IDD and co-occurring MH conditions. The Individual-level theme refers to the personal challenges that individuals with IDD and MH condition encounter as they transition from pediatric and child mental health services to the adult health and mental health systems of care. Respondents noted that some YYA with IDD and co-occurring MH conditions were not prepared or equipped to move from a pediatric physical and MH care model offering developmentally appropriate and family-centered care to an adult model of care that did not offer similar considerations… One responded stated *“They need training so they can learn (this may take many years) to navigate the medical field and have advocates to protect them from poor medical decisions.”*

### Self-advocacy

An additional important concept that emerged within the individual-level theme was self-advocacy. Self-advocacy refers to the ability of the young person with IDD to be able to speak up for themselves in terms of their interests, needs and preferences. Self-advocacy suggests that the individual is comfortable in asserting himself/herself in making choices and decisions. Knowledge of one’s rights is an important component of self-advocacy, and respondents indicated the importance of this, and need for training to support this, in the following quotes: *“Young people transitioning to adult healthcare and mental-healthcare need to know that they are in control of their own care plans and they have choices, but real-world consequences need to be informed, instructed and decisions need to be respected.”*

## Family-level Barriers

These barriers referred to problems that family members faced in assisting and supporting their children associated with transferring their care to adult health and MH care providers. Their responses were captured into two closely related subthemes: the need for family support and the need for family training.

### Need for Family Support

Respondents identified the need for family support during HCT, an element that was often missing, since families were accustomed to pediatric, child-centered services, but very unfamiliar with how to access and navigate adult serving systems and programs. As one respondent stated: *“Families don’t know how to navigate the system and support their needs.”* In addition, it was noted that additional challenges may impact a family’s ability to navigate the transfer to care and services: *“socioeconomic barriers impact caregivers’ ability to follow through.”*

### Need for Family Training

Another family-level barrier identified by respondents was the need for family training to prepare them for responsibilities related to transfer of care. As respondents noted, parents needed training to enable them to better assist and support their children with accessing transition and adult services. Respondents acknowledged that transitioning from child to adult MH services was a new challenge for all parents, including those who had been very involved with their child’s pediatric physical and mental health care, as stated: *“Many of the youth with IDD MH have parents that are very involved in their care, [but] preparing parents for this role with their adult children is often missing.”* Another respondent stated that a barrier to transition is that “*parents are not well-informed”* indicating a serious gap in family preparation and training by relevant systems.

## Provider-Level Barriers

Respondents identified a number of different types of provider-level barriers that were categorized by the following subthemes: lack of provider knowledge, lack of mental health providers or staff, and problems with mental health treatment. These subthemes are described in greater detail below.

### Lack of Provider Knowledge

Respondents noted one of the provider barriers was the lack of knowledge about the differences between IDD and mental health behavioral manifestations. That is, it was observed by respondents that adult providers may not have the necessary clinical skills to differentiate behaviors associated with the IDD diagnosis from those associated with having a MH condition. This dilemma, associated with diagnostic limitations has been referred to as “diagnostic overshadowing” (Hallyburton, 2022) wherein behaviors manifested by the individual were associated with the primary diagnosis of IDD rather than that of behaviors that may instead be symptoms of a separate MH condition. These comments reflect those observations:

*“Denial of acknowledgement of mental health conditions due to difficulty diagnosing, especially for non-verbal young people with IDD and mental health conditions.”; “Many families have reported that they are unable to secure mental health services for their adult child who has autism, because all of the child’s behaviors are blamed on autism.”*

Lack of adequate knowledge on providing services to young people with both IDD and MH conditions seemed to indicate the need for provider training. In response to the question “what other barriers exist for young people with IDD and mental health conditions in transitioning to adult mental health care, respondents shared: *“Lack of knowledge among mental health providers in understanding co-occurring diagnoses in people with intellectual and developmental disabilities.”* and *“ER staff and other first responders not trained in working with adults with ID.”*

### Lack of MH Providers or Staff

Respondents cited a lack of adequate workforce with experience providing services to young people with IDD and co-occurring MH conditions. A respondent shared this comment in this regard, *“[The]lack of specialists trained in this area is needed to address the health care of individuals with IDD and other mental health conditions [is a barrier to transition to MH care]. “Another* respondent cited “*high turnover”* as a contributor to the specialist workforce shortage problem. Other respondents noted that the lack of specialized expertise is a barrier to transition to MH care, as evidenced by this remark, *“Not enough people…are trained in treating patients who are nonverbal or have communication challenges.”*

### Problems with MH Treatment

Respondents also noted that there were problems associated with the treatment approach, such as providers attributing challenges or behaviors to the developmental disability and not the MH condition, and vice versa, and therefore individuals could not find help with either needs related to IDD or the co-occurring MH condition. As this respondent noted, “*Each ‘specialty’ wants the patient to deal with the other issues first and one is sent in a spiraling circular worm hole trying to find care anywhere.”*

Comorbid IDD and mental health problems present complexity, and specialists in either IDD or MH may approach this from only one lens, rather than a wholistic approach, which often seems to lead to treatment plans that are not adequate and/or may not fully meet the needs of these youth, As one respondent noted* “Many exhibit behavioral issues and providers are not flexible to meet their needs.”*

## System-of-Care-Level Barriers

The System-of-Care Level theme refers to the barriers that respondents identified that were inherent and problematic within systems of care that prevented the uninterrupted and coordinated the transfer of care from child to adult mental health services for YYA with IDD and co-occurring MH conditions. Four sub-themes emerged from this major theme and are described below:

### Problems with Access to Care

Mental health services for young people with IDD and co-occurring MH conditions are scarce and therefore difficult to locate and difficult to access due to long wait lists, as reported by multiple respondents. One respondent even shared there was a “*lack of MH services*.” This statement reflects repeated observations made by respondents. If services were available, respondents noted that there were wait lists that served to delay access to needed MH services in a timely manner. These statements were evidence of the problems in attempting to access services: “*[It} takes a long time to get in (when the law says it must not)” “Long waits when accessing MH services, if they can get them at all.”*

### IDD Diagnostic Bias and Labelling

Respondents reported that the primary diagnosis of IDD was a barrier to accessing mental health services. The IDD diagnosis was considered a disqualifier for service eligibility as this respondent’s statement indicates *“Autism appears to disqualify many individuals seeking mental health services.”* Given this service orientation, a respondent shared a policy perception that *“Adult Department of Mental Health (DMH) agencies ALWAYS state they don’t treat individuals with IDD, regardless of mental health.”* The policy directive created barriers in trying to access care as respondents noted that MH professionals referred young people back to IDD providers as responsible agents for services. Respondents offered these comments that reflected this perspective: “*Mental health points to the IDD services system and the IDD service systems points to Mental Health system and the person is caught in the middle.” “[A barrier to transition is the (Developmental disability service system)] bias and propensity to deny cases using excuse of primary diagnosis being mental health related vs. Autism or IDD.”*

### Language and Culture Barriers

Other system barriers identified by respondents were the language and cultural issues that were problematic in accessing MH services for young people with IDD. The limitations associated with not having language accessible resource information was evident in respondents’ comments such as, *“Lack of care in native language or explanation in Native Language.” “Lack of information/support available in threshold and plain language.”* The inadequacy of cultural competency amongst MH service providers serving culturally diverse populations was noted by respondents. This statement exemplifies those perspectives: *“…add in the lack of cultural competency and it's worse for people of color or [those] whose primary language is not English, [or] they have some other difference (sexual orientation or identity, religion, *etc*.)”.*

### Need for Resources Related to Social Services and Case Management

Respondents noted resources for young people with IDD who have MH conditions transitioning to adult care are limited. “*More assistance needed for young adults who can't access existing adult/youth in transition programs due to mental health and behavior problems.”* Other service needs included “*community resources for coordinated care and support”* as well as *“financial (assistance),”* and *“access to legal help.”* Though these resources would best be utilized on an individual and/or family level, the lack of these resources can be considered a systems-level issue, and advocacy to increase these resources must occur on a systems level as well.

## Societal-level Barriers: Discrimination and Stigma

### Provider and Systems Discrimination

Provider bias and systems discrimination were named by respondents as barriers that were characterized as pervasive throughout the system of care. The discriminatory practices, directed to young people of color, or based on MH diagnosis, were described. This respondent statement, captures that sentiment, “*Racial bias to care for certain groups that are not of color.”* Other responses indicated denial of services in a discriminatory manner, such as *“(the DD service system) has a bias and propensity to deny cases using the excuse of primary diagnosis being mental health related vs. autism or IDD.”*

See Table 3 for codes based on the social ecological model.

**Table 3:** Barriers for young people with IDD and mental health condition in terms of adult transition

Level	Example Themes	Illustrative Quotes
Individual	Self-advocacy	“Young people transitioning to adult healthcare and mental-healthcare, needed to know that they are in control of their own care plans and they have choices, but real world consequences need to be informed, instructed and decisions need to be respected.”
		“People with special needs…need training so they can learn (this may take many years) to navigate the medical field and have advocates to protect them from poor medical decisions.”
Family	Need for family support	“Families don’t know how to navigate the system and support their needs.”
	Need for family training	“Many of the youth with IDD MH have parents that are very involved in their care. preparing parent for this role with their adult children is often missing.”
Provider	Lack of knowledge of providers about difference between IDD and MH/need for provider training	“Denial of acknowledgement of mental health conditions due to difficulty diagnosing, especially for non-verbal young people with IDD and mental health conditions.”
		“Many families have reported that they are unable to secure mental health services for their adult child who has autism, because all of the child's behaviors are blamed on autism.”
		“Lack of knowledge among mental health providers in understanding co-occurring diagnoses in people with intellectual and developmental disabilities.”
		“ER staff and other first responders not trained in working with adults with ID.”
	Lack of MH providers or staff	“Lack of specialists trained in this area is needed to address the health care of individuals with IDD and other mental health conditions.”
		“High turnover.”
		“Not enough people who are trained in treating patients who are nonverbal or have communication challenges.”
	Problems with MH treatment	“Each “specialty” wants the patient to deal with the other issues first and one is sent in a spiraling circular worm hole trying to find care anywhere.”
		“Many exhibit behavioral issues and providers are not flexible to meet their needs.”
Systems of care	Problems with access to care	“lack of MH services
		“Takes a long time to get in (when the law says it must not)”
		“Long waits when accessing MH services, if they can get them at all.”
		“Autism appears to disqualify many individuals seeking mental health services.”
	Systems barriers: IDD diagnosis causing barrier to MH treatment; MH condition barrier to IDD resources	“Adult Department of Mental Health (DMH) agencies ALWAYS state they don't treat individuals with IDD, regardless of mental health.”
		“Mental health points to the I/DD services system and the I/DD service systems points to Mental Health system and the person is caught in the middle.”
		“(Developmental disability service system) bias and propensity to deny cases using excuse of primary diagnosis being mental health related vs. Autism or IDD
		“Lack of care in native language or explanation in Native Language.”
	Language and culture barriers	“…add in the lack of cultural competency and it's worse for people of color or whose primary language is not English, other they have some other difference (sexual orientation or identity, religion, etc.)”
		“Lack of information/support available in threshold and plain language.”
	Need for resources related to social services and case management	“More assistance needed for young adults who can't access existing adult/youth in transition programs due to mental health and behavior problems.”
		“…lack of community resources for coordinated care and support.”
		“(need for) financial (assistance), access to legal help
Society	Provider and systems discrimination	“Racial bias to care for certain groups that are not of color.”

## Discussion

The study was the first to our knowledge to elicit open ended responses from stakeholders that describe the barriers that individuals with IDD and IDD and co-occurring MH conditions face during the transition to adulthood. These barriers were stratified into the 5 SEM constructs: individual-level, family-level, provider-level, system of care-level and societal-level, each of which created challenges to successful transition to the adult system of care. The analysis revealed that the scope of barriers encountered create various challenges, which impede the transfer to adult health and MH care. Researchers have reported problems with accessing adult MH services due to differing eligibility criteria for adult MH services than for pediatric MH care (McNamara et al., 2014). In additions, individuals with IDD and co-occurring MH conditions may be “bounced back and forth” between agencies that address MH needs and those that serve developmental disabilities, with each one attributing responsibility to the other as having the service obligation. This may be due to differing eligibility criteria for services and supports through state departments of mental health versus state programs that serve individuals with IDD, such has home and community based-services (HCBS) waivers. However, despite policy changes that break down some of these divisions, such as states requiring health care plans to cover behavioral treatment for autism (L&M Policy Research, 2013), a recent study of US mental health facilities indicate that only 43 percent offer treatment for individuals with autism (Cantor et al., 2020). We heard multiple times that it appears that an individual’s MH condition may appear to disqualify them from obtaining DD services, and likewise, and individuals with IDD may then be disqualified from receiving appropriate MH services.

Stakeholders indicated that lack of MH providers and staff, both in number and who have appropriate training, is a significant barrier. This barrier was identified previously in a number of studies and review papers. In addition, high turnover of MH staff was identified by survey participants as a barrier to transitioning to adult MH care serviced for individuals with IDD, consistent with a known concerning trend of high turnover rates of MH providers (Beidas et al., 2016; Johnson et al., 2018), with cited reasons in the literature including due to high levels of burnout (Beidas et al., 2016; Johnson et al., 2018). As found in this study and reported in prior studies, significant challenges exist for individuals with IDD and co-occurring MH conditions to access providers with the knowledge and clinical expertise to provide needed services (Auerbach et al., 2018; Broad et al., 2017; Franklin et al., 2019; Hendrickx et al., 2020; Pinals et al., 2022; Pouls et al., 2022; Reale & Bonati, 2015; Shady et al., 2022; Signorini et al., 2018).

Findings pertaining to family-level barriers revealed the importance of family involvement and the challenges they faced in navigating new systems of care. Our analysis of data revealing the self-advocacy competencies that individuals with IDD and IDD and co-occurring MH conditions should obtain, particularly the increase in knowledge that is required as their care is transferred to adult health and MH systems of care, is an emergent finding not previously reported in the literature. Previous reports have focused on deficits and limitations, such as communication challenges of consumers that are perceived as problematic as they transfer care into adult systems. Self-advocacy is a life-span issue that warrants ongoing attention and support by providers and their families. Promoting self-advocacy is a component of care that needs to be integrated into all aspects of service provision. It is important for service providers to encourage active engagement in clinical encounters, supported decision-making and person-centered care that is based on their needs, interests and preferences. Coordination of care and referrals to community-based programs that foster learning self-advocacy skills, promote community inclusion and independence and access to peer networks will assist with the achievement of developmental milestones associated with adulthood, including making independent decisions about health care, education, employment, relationships, and independent living.

Lastly, the societal barrier of provider and system discrimination has been reported extensively in the literature. As respondents shared in this survey, this barrier is widespread throughout the system. Greater disparities exist for individuals with IDD and co-occurring MH conditions in accessing health care transition services than for individuals with either condition, alone with either condition alone w (Cheak-Zamora et al., 2013; Leeb et al., 2020; Munir, 2016; Zablotsky et al., 2020). Our study highlighted specific issues that individuals with IDD and co-occurring MH face, such as being excluded from IDD service systems due to their MH condition, and simultaneously excluded from MH care systems due to their IDD. This is a form of discrimination, as it represents exclusion based on a characteristic, and makes transition to adult services increasingly problematic.

## Limitations

Information on each survey participant was limited, including the type of provider and type of advocate. We did not clearly delineate if the respondent identified with a diagnosis of IDD, or how many of the “advocates” who responded were “self-advocates” which is generally assumed to mean an individual with IDD, versus family or professional advocates, as all of these categories was grouped as “disabilities advocate” as a respondent type in our survey. In addition, as the majority of our respondents indicated that they primarily serve populations in California, this makes our findings less generalizable to the rest of the United States or internationally. Furthermore, though our mailing list included both internal and external stakeholders, we likely had more responses from faculty and staff internal to our UCEDD which can impact generalizability as well. However, issues stated by our respondents, such as lack of adequate preparation for transition, need for increased support and case management, and workforce issues have been similarly expressed in studies both outside our state and country (Cleverley et al., 2020; Culnane et al., 2021; Cvejic & Trollor, 2018). In addition, we analyzed open-ended responses from a written survey, and not an oral interview, thus responses may have been limited and interviews or focus groups may have yielded deeper and richer information. Lastly, as our question “what other barriers exist for young people with IDD and mental health conditions in transitioning to adult mental health care?” did not specify whether respondents should comment on the pediatric or adult system, responses were likely mixed in their reference to pediatric and adult care issues. However, in general, the findings highlighted the need for more and improved training of individuals and families to prepare them for the transition from pediatric to adult care and systems, which should start in pediatric settings. In addition, the lack of appropriately trained providers, and barriers related to conflicts between the IDD and MH systems bridge pediatric and adult systems of care. These findings indicate a major need to embark on systems changes, to minimize the “bouncing back and forth” between the IDD and MH systems for individuals with IDD to obtain care, and to develop the workforce so that a greater number of professionals have appropriate training and comfort to care for individuals with IDD and co-occurring MH conditions.

## Conclusion

The social ecological model indicated a need for improvement in availability and delivery of services at the individual, family, provider, systems of care, and society levels to better support transition of pediatric to adult health and MH care for YYA with IDD and co-occurring MH conditions. Suggestions for improvement could be organized into these SEM levels as well. On the individual level, there could be improved coaching of YYA regarding how to talk to their health and MH care providers about their needs and encouraging use of health passports (Dharampuriya & Abend, 2022). On a family level, involving parents with lived experience as “navigators” may help families approach a future vision for their children with IDD in a supportive way (Mirzaian et al., 2023). On a provider level, there is a clear need to increase training to address needs of individuals with both IDD and co-occurring MH conditions (Cantor et al., 2020), and on a systems level, improving integration between systems that support MH conditions and systems that support individuals with IDD are imperative. Lastly, on a societal level, efforts to decrease discrimination and explicit and implicit bias against individuals with disabilities, particularly those with IDD and co-occurring MH conditions, and from racial groups that also face higher levels of discrimination, is imperative. The issues raised in this investigation are relevant and timely to initiate calls to action to improve health and MH care transitions for individuals with IDD with or without co-occurring MH conditions.

Future research should investigate how policies including payment systems for treatments and supports for MH diagnoses versus IDD diagnoses are structured and how this impacts both delivery and transition of care. In addition, it is apparent that it necessary to build a provider workforce to care for individuals with IDD and co-occurring MH conditions in adulthood through increased training as well as potential reform of payment models to make this care feasible, and even potentially incentivized. In addition, evidence-based methods of training and preparing both families and individuals to face the difficult transition from pediatric to adult health and mental health care for YYA with IDD and co-occurring MH conditions will be important to continue these efforts.
